# The contribution of transcranial magnetic stimulation in the functional evaluation of microcircuits in human motor cortex

**DOI:** 10.3389/fncir.2013.00018

**Published:** 2013-02-13

**Authors:** Vincenzo Di Lazzaro, Ulf Ziemann

**Affiliations:** ^1^Institute of Neurology, Campus Biomedico UniversityRome, Italy; ^2^Fondazione Alberto Sordi - Research Institute for AgeingRome, Italy; ^3^Department of Neurology and Stroke, Hertie-Institute for Clinical Brain Research, Eberhard Karls University TübingenTübingen, Germany

**Keywords:** transcranial magnetic stimulation, motor cortex, pyramidal neuron, GABAergic neuron, corticospinal tract

## Abstract

Although transcranial magnetic stimulation (TMS) activates a number of different neuron types in the cortex, the final output elicited in corticospinal neurones is surprisingly stereotyped. A single TMS pulse evokes a series of descending corticospinal volleys that are separated from each other by about 1.5 ms (i.e., ~670 Hz). This evoked descending corticospinal activity can be directly recorded by an epidural electrode placed over the high cervical cord. The earliest wave is thought to originate from the *direct* activation of the axons of fast-conducting pyramidal tract neurones (PTN) and is therefore termed “D” wave. The later waves are thought to originate from *indirect*, trans-synaptic activation of PTNs and are termed “I” waves. The anatomical and computational characteristics of a canonical microcircuit model of cerebral cortex composed of layer II and III and layer V excitatory pyramidal cells, inhibitory interneurons, and cortico-cortical and thalamo-cortical inputs can account for the main characteristics of the corticospinal activity evoked by TMS including its regular and rhythmic nature, the stimulus intensity-dependence and its pharmacological modulation. In this review we summarize present knowledge of the physiological basis of the effects of TMS of the human motor cortex describing possible interactions between TMS and simple canonical microcircuits of neocortex. According to the canonical model, a TMS pulse induces strong depolarization of the excitatory cells in the superficial layers of the circuit. This leads to highly synchronized recruitment of clusters of excitatory neurons, including layer V PTNs, and of inhibitory interneurons producing a high frequency (~670 Hz) repetitive discharge of the corticospinal axons. The role of the inhibitory circuits is crucial to entrain the firing of the excitatory networks to produce a high-frequency discharge and to control the number and magnitude of evoked excitatory discharge in layer V PTNs. In summary, simple canonical microcircuits of neocortex can explain activation of corticospinal neurons in human motor cortex by TMS.

## Introduction

Transcranial magnetic stimulation (TMS) and transcranial electrical stimulation (TES) can activate the human brain through the intact scalp (Merton and Morton, 1980; Barker et al., 1985). The effects induced by TMS or TES can be measured as motor evoked potentials (MEPs) by electromyography and/or as TMS-evoked EEG potentials (TEPs) by electroencephalography (EEG) (Ilmoniemi et al., 1997). However, we still have limited information about which neurones are activated by TMS and about the mechanisms of interaction of TMS effects with ongoing neuronal activity in cortical circuits. One way of assessing the effects of TMS is to directly record the synaptic activity evoked by stimulation of those cortical areas that produce a measureable readout, such as the primary motor cortex (M1). This was done first in cats and monkeys (Adrian and Moruzzi, 1939; Patton and Amassian, 1954) and more recently in conscious humans (Boyd et al., 1986). Cumulatively, these studies showed that the output produced by M1 stimulation is surprisingly stereotyped both in animals and in humans.

## Animal studies

Direct recording from the pyramidal tract in cats and non-human primates with an electrode placed in the medullary pyramid or on the dorsolateral surface of the cervical spinal cord showed in response to a single electrical stimulus applied to M1 a series of high-frequency descending waves (Adrian and Moruzzi, 1939; Patton and Amassian, 1954; Kernell and Chien-Ping, 1967; Amassian et al., 1987). The earliest wave that persisted after cortical depression and even after cortical ablation was thought to originate from the *direct* excitation of the axons of fast-conducting pyramidal tract neurones (PTN) and was therefore termed “D” wave. The later waves evoked by cortical stimulation required the integrity of the cortical gray matter, and were thought to originate from *indirect*, trans-synaptic activation of PTNs and were therefore termed “I” waves (Patton and Amassian, 1954). Recordings from individual PTN axons showed that both a D and subsequent I wave discharge can be elicited in a given corticospinal axon (Kernell and Chien-Ping, 1967). Since the intervals between subsequent these discharges are in the range of 1.5 ms, this means that corticospinal cells are capable of firing at rates of ~670 Hz.

## Human studies

More recently, direct recording of the activity evoked by transcranial stimulation was performed in humans. The initial studies were performed in anaesthetized patients during surgery (Boyd et al., 1986; Berardelli et al., 1990; Thompson et al., 1991; Burke et al., 1993). These studies showed that both TES and TMS could evoke a series of waves traveling down the corticospinal tract. However, the level of anaesthesia had pronounced depressant effects on the recruitment of descending waves, impeding a characterization of the physiology of the M1 output elicited by transcranial stimulation. A few years later, Kaneko and colleagues (1996) and Nakamura and co-workers (1996) recorded for the first time the descending volleys evoked by transcranial stimulation in conscious human subjects from epidural electrodes implanted chronically in the spinal cord for the relief of pain. From 1998 onwards, Di Lazzaro and co-workers have performed an extensive series of studies using the same approach (see Di Lazzaro et al., 2012 for a review). These studies showed that the threshold for the activation of the different components of the descending volley is substantially different (Di Lazzaro et al., 2012). Around MEP threshold intensity, TES evokes a short latency wave that is not modifiable in amplitude or latency by changes in motor cortical excitability (such as voluntary contraction) and is believed to originate from direct excitation of corticospinal axons in the subcortical white matter at some distance from the cell body (Di Lazzaro et al., 1998a,1999b). At low intensity, TMS using a focal figure-of-eight stimulating coil and a monophasic posterior to anterior (PA) induced current in the brain evokes a single descending wave with a latency about 1 ms longer than the D-wave, that is thought to originate from the activation of monosynaptic cortico-cortical connections projecting onto corticospinal neurones (Di Lazzaro et al., 1998a,b). This descending wave produced by indirect trans-synaptic activation of PTNs has been termed I1 wave. At higher stimulus intensities later volleys appear: these are termed late I-waves and are thought to originate from repetitive discharge of PTNs through reverberating activation in a microcircuit of highly connected excitatory cells (Di Lazzaro et al., 2012). A further increase of TMS intensity leads to a direct excitation of the PTN axons resulting in a D-wave (Di Lazzaro et al., 1998a). When the orientation of the figure-of-eight coil is changed, so that monophasic currents in the brain are induced in a lateral to medial (LM) direction, TMS recruits a D-wave even at MEP threshold intensity (Di Lazzaro et al., 1998a).

When the direction of the stimulating current is reversed from PA to an anterior-posterior (AP) direction, the descending volleys have slightly different peak latencies and/or longer duration than those induced by PA stimulation, and the order of recruitment of the descending corticospinal waves may change with late I-waves already evoked at TMS intensity close to MEP threshold (Di Lazzaro et al., 2001). These findings provide evidence that PA, AP, and LM TMS activate different populations of cortical neurones/axons in the motor cortical circuitry, or the same populations but at different sites.

## Cortical networks activated by single pulse TMS

The circuit generating the I1 wave and that generating the late I-waves show different sensitivity to several interventions. The late I-waves but not the I1 wave are (1) depressed by enhancement of neurotransmission through the inhibitory gamma-aminobutyric type A receptor (GABAAR) by benzodiazepines (Di Lazzaro et al., 2000); (2) depressed by paired-pulse TMS protocols testing intracortical inhibition (Nakamura et al., 1997; Di Lazzaro et al., 1999a, 2002a; Tokimura et al., 2000); and (3) enhanced/depressed by various repetitive TMS protocols that induce long term potentiation-like and long term depression-like plasticity in the human brain (for review, Di Lazzaro et al., 2010).

Several models have been proposed to explain the origin and nature of the descending corticospinal waves evoked by TMS in humans (Creutzfeldt et al., 1964; Amassian et al., 1987; Phillips, 1987; Day et al., 1989; Sakai et al., 1997; Ziemann and Rothwell, 2000). In 1987, Amassian and co-workers (1987) suggested that I-waves were produced by a periodic bombardment of corticospinal cells through chains of interneurons with fixed temporal characteristics. An alternative model, firstly proposed by Creutzfeldt et al. (1964) and further developed by Phillips (1987) and Ziemann and Rothwell (2000), suggests that single-pulse motor cortex stimulation produces strong and synchronized depolarization of many corticospinal cells and/or interneurons, which leads to oscillatory activity and repetitive discharge of these cells as a consequence of their intrinsic membrane properties. The models above do not explain the observation that it is possible to recruit late I-waves in isolation or to suppress late I-waves with no effect on the I1 wave with various single and paired TMS protocols (Di Lazzaro et al., 2008). These findings suggest that early and late I-waves are generated through at least partially independent cortical circuits. A model derived from the one proposed by Amassian and co-workers suggests that repetitive I-wave discharge is generated by activation of independent chains of interneurons, each responsible for generating a different I-wave (Day et al., 1989; Sakai et al., 1997). This model would better explain the dissociable modulation of the different components of the corticospinal volley in various TMS protocols of motor cortex stimulation. However, this model is difficult to reconcile with the remarkably stable interpeak interval between early and late I-waves demonstrated both in animal and human epidural recordings, moreover, it does not explain why, in the paired-pulse facilitation experiments there is no summation of effects at intermediary intervals between the peaks (0.5–0.9, 1.6–2.2. and 3.0–4.0 ms) (Tokimura et al., 1996; Ziemann et al., 1998). None of the above models does fully explain all of the up-to-now known properties of the D- and I-waves. Moreover, all of these models were built on empirical observations provided by MEP recordings and epidural recordings of the descending corticospinal volleys but not on an established model of cortical circuit organization. A model incorporating the detailed anatomy of M1 was proposed by Esser and co-workers (2005) who aimed at explaining the effects of TMS on motor cortical circuits by constructing a large-scale model including the thalamo-cortical system and a three-layered motor cortex with more than 30,000 neurons and more than 5 million intra- and inter-layer synaptic connections. Those authors suggested that the sequence of events underlying the generation of I-waves is best approximated as a combination of intrinsic neuronal properties (model of a neural oscillator) and interactions between circuits of inhibitory and excitatory interneurons (Esser et al., 2005). This work is the so far most elaborated attempt to model the occurrence of I-waves and the modeled responses to simulated TMS pulses were consistent with the observed epidural recordings of the corticospinal volley in various single and paired-pulse experiments, including the frequency, timing, dose response, and pharmacological modulation of I-waves. However, the model is complex and required a number of constraints such as the assumption of an *ad hoc* refractory mechanism to explain the frequency of I-waves.

**Figure 1:**
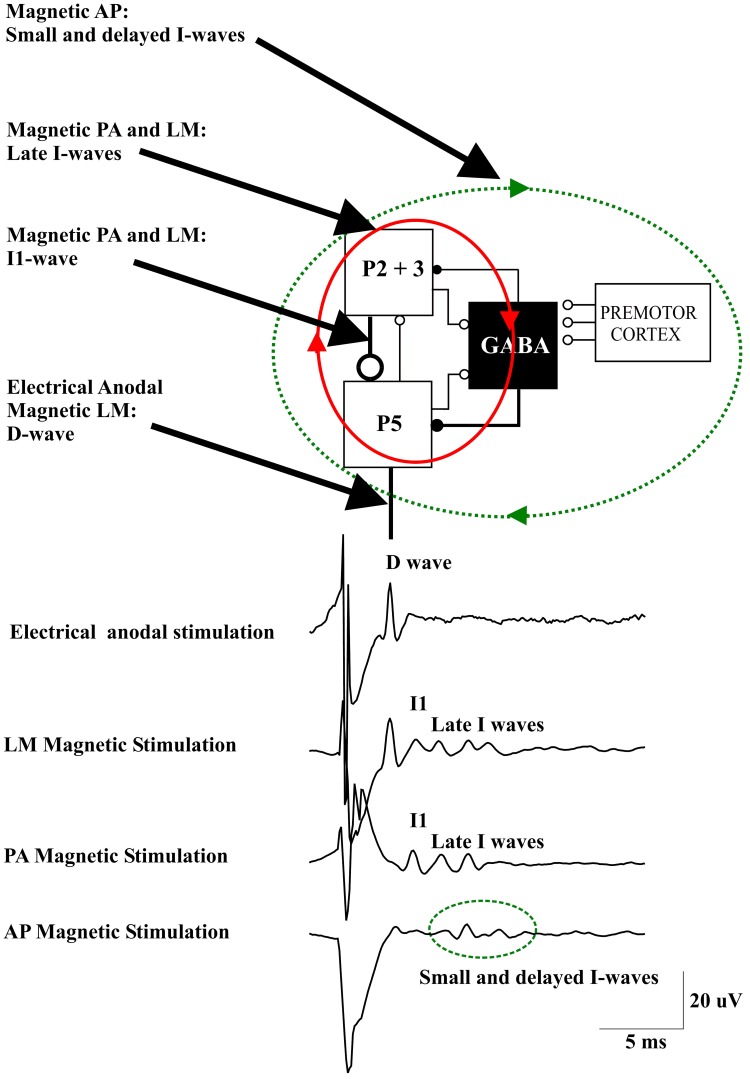
**A schematic view of the model of corticospinal volley generation based on canonical cortical circuit proposed by Douglas et al. (1989).** This model includes the superficial population of excitatory pyramidal neurons of layers II and III (P2-P3), the large pyramidal tract neurons in layer V (P5), and the inhibitory GABA cells [modified from Figure 1.14 in “The Synaptic Organization of the Brain” (Shepherd, 2004)]. Electrical anodal stimulation activates the axons of P5 cells evoking a D wave. Magnetic stimulation with a latero-medial (LM) induced current in the brain produces a direct activation of the axons of corticospinal cells evoking the D wave followed by an I1 wave produced by monosynaptic activation of P5 cells by the axons of superficial pyramidal neurons, at high intensities it also produces a recurrent activity in the circuit composed of the layer II and III and layer V pyramidal neurons together with their connections with local GABAergic interneurons (red ellipse and arrows) evoking late I-waves. Magnetic stimulation with a posterior-anterior (PA) induced current in the brain evokes the I1 wave and, at higher intensities, late I-waves. Magnetic stimulation with an anterior-posterior (AP) induced current in the brain recruits small and delayed descending volleys with slightly different peak latencies and longer duration than those seen after posterior to anterior magnetic stimulation. It is proposed that this more dispersed descending activity is produced by a more complex circuit (green dotted ellipse and arrows) that might include cortico-cortical fibers originating from the premotor cortex and projecting upon the motor cortex circuits generating the I-waves.

Recently, Di Lazzaro and co-workers (2012) evaluated whether the properties of the descending waves recorded in conscious humans after transcranial stimulation can be explained by an interaction of transcranial stimuli and the cortical circuits as characterized by an established simple anatomical model. To this end, they used the basic “canonical” microcircuit of cerebral cortex proposed by Douglas et al. (1989) that can be applied to all neocortex. This model includes the superficial population of excitatory pyramidal neurons of layers II and III (P2-P3), the large PTNs in layer V (P5), and inhibitory GABAergic cells (Figure 1). It was proposed that this represents the minimum architecture necessary for capturing the most essential cortical input-output operations (Douglas et al., 1989; Shepherd, 1998). The canonical microcircuit of neocortex has a number of important characteristics that have been experimentally validated by intracellular recordings in cat visual cortex (Douglas and Martin, 1991). First, activation of the cortex induces a sequence of excitation and inhibition in every neuron. Second, the thalamic input does not provide the major excitation arriving at any neuron. Instead the intracortical excitatory connections provide most of the excitation (Douglas et al., 1989). The lowest intensities of anodal TES (anode over the M1 hand area, cathode over vertex) recruits an early descending volley, which has a latency of 2–2.6 ms when recorded from the high cervical cord (Di Lazzaro et al., 1999b). In humans, this latency suggests direct excitation of PTN axons just below the gray matter and, hence, this wave is referred to as a D wave. In the canonical circuit model, this wave might originate from direct excitation of P5 axons (Figure 1).

Using a focal coil and a monophasic waveform stimulus inducing a PA current across the central sulcus in the brain, the lowest threshold volley occurs at a latency 1.0–1.4 ms longer than the volley recruited by anodal TES (Di Lazzaro et al., 1998a). The axons of the more superficial pyramidal neurons (P2, P3) are conceivably the most excitable neural elements to transcranial stimulation, due to their superficial location close to the stimulating coil. They also represent the main source of input to PTNs (Anderson et al., 2010). According to the canonical microcircuit of cerebral cortex, these neurons have a monosynaptic excitatory connection with the large PTNs of layer V (P5) (Figure 1). Thus, the I1 wave might originate from monosynaptic activation of the P5 cells by direct excitation of axons of P2 and P3 cells (Figure 1). The difference in latency between the D-wave and the I1 wave of about 1 ms is appropriate for a monosynaptic activation of P5 cells originating from presynaptic axons. The excitatory postsynaptic potential (EPSP) in P5 cells is followed by an inhibitory postsynaptic potential (IPSP) after one additional synaptic delay because the connection between superficial excitatory neurons and P5 cells is disynaptic as it involves the relay via an inhibitory interneuron (Figure 1). Thus, the monosynaptic EPSP leading to the I1-wave is not influenced by the later arriving TMS-induced inhibitory GABAAergic input.

The I1 wave increases in size, and is followed by later volleys as the intensity of stimulation increases. The interpeak interval between I-waves is about 1.5 ms, which indicates a discharge frequency of about 670 Hz. According to the canonical microcircuit, the P5 cells have excitatory monosynaptic reciprocal connections with layer 2 and 3 pyramidal neurons and interneurons. Thus, these cells are activated after P5 discharge and they will in turn re-activate the P5 cells (Figure 1). The mean transmission delay between layer 2–3 neurons and layer 5 pyramidal neurons is about 1.5 ms. With a synaptic delay of 1.5 ms, computational models of networks of highly connected excitatory and inhibitory neurones predict a peak of activity of 667 Hz (Douglas et al., 1989) that corresponds to the I-wave frequency. This high frequency activity might be produced by the recruitment of fully synchronized clusters of excitatory and inhibitory neurones (Douglas et al., 1989). The interpeak latency of the I-wave discharge is too short to be explained by the refractory period of excitatory and inhibitory interneurons (Brunel and Wang, 2003). However, it should be considered that single-cell discharge rates are typically much lower than those of neuronal networks. Computational models of spiking neurons have shown that single pyramidal cells may fire only once in every 15–20 cycles of the population activity (Brunel and Wang, 2003). Synchronization of strongly interconnected groups of neurons that fire with millisecond precision may emerge in neuronal networks (Anderson et al., 2010). It can be assumed that with increasing intensity of the TMS pulse the duration of the induced neuronal activity increases, and that this goes along with an increasing number of recruited I-waves.

The model of I wave generation based on the canonical microcircuit model of neocortex proposes a circuit for the I1 wave generation represented by the monosynaptic excitatory connections between P2 and P3 cells and P5 cells and a network for the late I-waves that includes the same cortical elements together with their connections with local GABAAergic interneurons (Figure 1). This model can explain the fixed periodicity of the later I-waves and the close relationship between stimulus intensity and I-wave number.

Interestingly, the I-wave activity recorded after TMS resembles closely the 600 Hz EEG components recorded from the scalp in humans (Curio et al., 1994) and directly from cerebral cortex in monkeys (Baker et al., 2003) after electrical stimulation of peripheral nerves. Thus, it appears that high frequency discharge at around 600 Hz is a stereotyped response of pyramidal neurons in response either to cortical or to peripheral nerve stimulation.

The changes observed in I wave number and magnitude after manipulation of cortical excitability might also be explained by this model. According to the proposed model, the GABAAergic interneurons are involved only in the control of the late I-waves but not the I1 wave. This is in agreement with the changes observed in I waves after pharmacological enhancement of inhibitory GABAAergic activity through benzodiazepine administration. Lorazepam results in a pronounced suppression of the late I-waves while there is no change in the I1 wave (Di Lazzaro et al., 2000). This could be explained by an increase in IPSP amplitude in the P5 cells that prevents or limits the re-activation of P5 cells through the P5—P2/3 microcircuit. On the other hand, an increase of P5 cell excitability would result in an increase in the number and magnitude of late I-waves. It has been shown that maximum voluntary contraction can substantially increase the amplitude of all I waves including the I1 wave (Di Lazzaro et al., 1998b). The increase in size of the I1 wave suggests a direct increase in excitability of PTNs. The large effect on the size of the descending corticospinal waves is not paralleled by a comparable effect on the threshold for evoking recognizable descending activity after TMS (Di Lazzaro et al., 1998b). This suggests that the neural elements activated by PA TMS have a relatively constant threshold. The likely explanation for this dissociation is that magnetic stimulation activates axons projecting upon PTNs at some distance from the PTN cell body so that the threshold is unaffected by synaptic activity. According to the canonical microcircuit model the activated axons belong to the superficial P2 and P3 cells. At higher TMS intensities an earlier wave of small amplitude appears. This wave has the same latency as the D-wave evoked by anodal TES at threshold, and as for the TES evoked D-wave, it is proposed to be generated by direct excitation of P5 axons. The D-wave can be evoked even at low stimulus intensity by changing the direction of the induced current in the brain to LM orientation (Di Lazzaro et al., 1998a) (Figure 1).

Changing the parameters of the stimulus may lead to a more complex output of M1. When reversing the direction of the induced current in the brain from the usual PA direction to AP, smaller descending volleys are evoked with slightly different peak latencies and/or longer duration than those seen after PA stimulation (Figure 1) (Di Lazzaro et al., 2001). The peak latencies of the I-waves evoked by AP stimulation are delayed by 0.2–0.7 ms when compared with those evoked by AP stimulation. These smaller and delayed late I-waves are similar to those recorded in monkeys elicited by ventral premotor cortex stimulation (Shimazu et al., 2004). These authors showed that stimulation of the ventral premotor cortex evokes smaller and later I-waves than those evoked by M1 stimulation and suggested that this activity may be mediated by cortico-cortical inputs to M1 impinging onto M1 interneurons generating late I-waves (Shimazu et al., 2004). A similar mechanism might explain the more dispersed and delayed I-wave activity evoked by AP magnetic stimulation in humans.

## Non-focal stimulation of the brain (circular coil)

In anaesthetized human subjects, Burke and co-workers (1993) showed that, using a circular coil centred over the vertex and recording from the spinal cord, the D wave is the component with lowest threshold. Slightly different results were obtained in conscious subjects (Di Lazzaro et al., 2002b). The earliest wave evoked by a circular coil centred over the vertex had a latency that was ~0.2 ms longer than the D-wave elicited by anodal TES or LM magnetic stimulation. Either this wave or a wave corresponding to the I1 wave evoked by PA magnetic stimulation was the lowest threshold volley evoked by a circular coil in different individuals. At suprathreshold intensities later waves could be recruited: in some cases, these waves had latencies that were outside the periodicity of I-waves evoked by PA magnetic stimulation. A maximum voluntary contraction increased the amplitude of all descending volleys including the earliest (D) wave. These data show that there are major differences between non-focal stimulation of the M1 hand area with a circular coil and focal stimulation with a figure-of-eight coil. Non-focal stimulation is more likely to evoke a D wave than PA focal stimulation. Moreover, this early (D) volley recruited by non-focal stimulation is facilitated by voluntary contraction. Together with the slightly longer latency this suggests that it is initiated closer to the cell body of the PTNs than the conventional D wave evoked by anodal TES or LM magnetic stimulation, perhaps at the axon hillock rather than at some distance down the axon. Such a proximal D-wave is also evoked by focal TMS using a biphasic current waveform (Di Lazzaro et al., 2002b).

## Cortical networks activated by paired pulse TMS

A variety of different methods have been introduced to explore the connections within M1, or the connections to M1 from other regions of the central nervous system. Different paired-pulse TMS protocols employ a conditioning stimulus either above or below MEP threshold intensity, at various interstimulus intervals (ISIs) to the test stimulus, and delivered either through the same stimulating coil for exploration of circuitry within M1, or through another stimulating coil for exploration of cortico-cortical connections to M1 [for review, (Ziemann and Hallett, 2007)]. Furthermore, other protocols employ an electrical conditioning stimulus applied to a peripheral nerve.

A subthreshold magnetic conditioning stimulus given through the same coil as the test stimulus can suppress a MEP evoked by a later suprathreshold test stimulus if the ISI between the stimuli is 5 ms or less: this phenomenon has been termed short-interval intracortical inhibition (SICI) (Kujirai et al., 1993). On the other hand, with ISIs from 10 to 25 ms, MEP facilitation is observed: this is known as intracortical facilitation (ICF) (Kujirai et al., 1993; Ziemann et al., 1996b).

**Figure 2:**
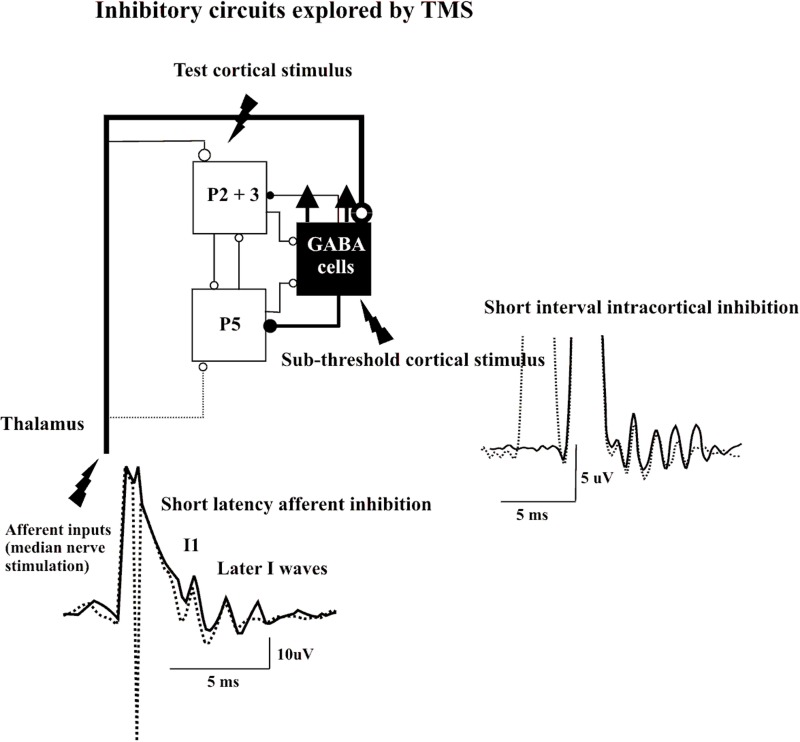
**Epidural volleys evoked by test magnetic stimulus alone (solid traces) and by test magnetic stimulus preceded by a subthreshold conditioning stimulus at 3 ms interstimulus interval (Short Interval Intracortical inhibition dotted trace) or by test magnetic stimulus preceded by a peripheral nerve stimulation to the median nerve at the wrist (Short Latency Afferent Inhibition dotted trace).** Each trace is the average of 10 sweeps. The test stimulus activates the axons of pyramidal neurons of layers II and II (P2 and P3) that in turn activate pyramidal neurons of layer V (P5) and the GABA cells projecting upon the layer V pyramidal cells evoking multiple descending waves. In the short interval intracortical inhibition protocol a clear suppression of the late corticospinal volley is evident when test magnetic stimulus is preceded by the conditioning subthreshold stimulus. It is proposed that the conditioning stimulus enhances selectively the excitability of the GABAergic connections with a suppression of the late I waves. In SAI protocol clear suppression of the latest corticospinal volley is evident when test magnetic stimulus is preceded by the peripheral nerve conditioning stimulus. It is proposed that the peripheral nerve stimulation enhances the excitability of the GABAergic cells through the activation of thalamocortical projections.

Direct epidural recordings of the descending corticospinal volleys demonstrated the cortical origin of SICI (Di Lazzaro et al., 1998c). A subthreshold conditioning stimulus that alone does not evoke descending corticospinal activity produced significant suppression of late I-waves if the ISI to the subsequent suprathreshold test stimulus was between 1 and 5 ms. In contrast, the I1 wave was virtually unaffected in the SICI protocol. Kujirai and colleagues (1993) originally suggested that SICI represents GABAAergic inhibition, and supportive evidence was provided for this contention by pharmacological experiments that showed an increase in SICI by lorazepam, a positive allosteric modulator at the GABAAR (Ziemann et al., 1996a). This hypothesis was further supported by the observation that administration of lorazepam increases the inhibition of the late I-waves but not the I1 wave in the SICI protocol (Di Lazzaro et al., 2000). Because conditioning stimulation that is subthreshold for the activation of PTNs produces SICI, this from of inhibition probably originates pre-synaptically to these cells. Since the I1 wave remains unaffected, it is unlikely that the subthreshold conditioning stimulus modifies the response of the PTNs to the excitatory inputs but enhances selectively GABAAergic neurotransmission leading to suppression of the late I-waves according to the canonical microcircuit model. This is consistent with TMS experiments that indicated that the SICI circuitry has a lower excitation threshold than the excitatory circuits in M1 (Kujirai et al., 1993; Ziemann et al., 1996b; Ilic et al., 2002). It should be considered that the functional characteristics of the inhibitory networks have a pronounced tendency to synchronize through axonal interconnections between GABAAergic cells, a property that increases their efficiency over excitatory networks (Hasenstaub et al., 2005). Thus, it can be speculated that subthreshold depolarization of the superficial pyramidal cell axons produces a short-term facilitation of GABAAergic neurotransmission that is expressed as significant late I-wave suppression in response to the test stimulus (Figure 2).

The mechanisms of ICF elicited by paired pulse stimulation at ISIs of 10–25 ms are more complex and less well understood. Epidural recordings showed that there was no significant change in the amplitude or number of descending corticospinal waves in the ICF protocol in the presence of a significant MEP facilitation (Di Lazzaro et al., 2006; Ni et al., 2011). One possible explanation for this dissociation is that ICF results from the recruitment of circuits separate from those involved in I-wave generation (Di Lazzaro et al., 2006): according to the canonical microcircuit model these could be the same circuits that are being activated by AP magnetic stimulation. These circuits might be long-range connections (e.g., from ventral premotor cortex) that might not be activated by single pulse PA stimulation but might be recruited by paired pulse stimulation. However, since the additionally evoked activity is more dispersed, it may not be evident in the epidural records in the presence of clear I waves. The activation of long-range connections originating from remote areas is also suggested by the strong dependence of ICF upon the direction of the conditioning current in the brain, a phenomenon not observed for SICI (Ziemann et al., 1996b). The reason why ICF occurs at a specific range of ISIs of 10–25 ms is still unclear.

Different paired cortical stimulation protocols use suprathreshold conditioning stimulation. These protocols may either result in facilitation or inhibition of the MEP and the descending corticospinal volley depending on the ISI. The facilitation observed at short ISIs is referred as short interval intracortical facilitation (SICF) (Tokimura et al., 1996; Ziemann et al., 1998). If two stimuli are given at an intensity at or slightly above active MEP threshold, then MEP facilitation can be observed at discrete ISIs of around 1.5, 3.0, and 4.5 ms, corresponding to the intervals between the I1 wave and the following I-waves. According to the canonical microcircuit model, this facilitatory interaction between the two pulses might be produced because the peaks of the I-waves evoked by the two stimuli are in phase and the input related to the second stimulus arrives during epochs of increased firing probability following the first stimulus. Moreover, the combined effect of the two stimuli in producing recurrent excitatory activity, when the peaks of the I-waves are in phase, might reinforce the synchronization of cortical networks prolonging their activity and evoking additional I-waves. Epidural recordings of the descending corticospinal volleys from the spinal cord demonstrated the interaction on individual I-waves very clearly (Di Lazzaro et al., 1999c). An interaction resembling that observed using the SICF protocol has also been reported in cortical cell cultures. The effects produced by stimuli phase-locked to the ongoing rhythmic activity in the network are phase specific: stimuli applied at the peak of the rhythmic activity have a facilitatory effect while stimuli applied at the trough produce inhibitory effects (Stegenga et al., 2010).

A suprathreshold conditioning stimulus suppresses a MEP to a later suprathreshold stimulus if the ISI is 100 ms or longer, this phenomenon has been termed long-interval intracortical inhibition (LICI) (Valls-Sole et al., 1992). The recording of corticospinal volleys in this paired-pulse paradigm showed that later I waves are reduced at ISIs of 100 ms, but the I1 wave remains unaffected (Nakamura et al., 1997; Chen et al., 1999; Di Lazzaro et al., 2002a). Because of its duration, it is believed that LICI is mediated by slow IPSPs mediated by the GABAB receptor (Douglas and Martin, 1991). This received direct support by the finding that baclofen, a specific GABAB receptor agonist, increases the magnitude of LICI (McDonnell et al., 2006). In analogy with SICI, the inhibition observed at 100 ms ISI originates from a selective suppression of the recurrent activity producing later I-waves with no effect on the I1 wave that, accordingly to the canonical circuit model, originates from monosynaptic excitatory connections not modulated by GABAergic connections.

A short latency afferent inhibition (SAI) of MEPs in hand muscles is produced by conditioning the cortical magnetic stimulus with electrical stimulation of sensory peripheral nerves of the hand (Tokimura et al., 2000). This phenomenon of SAI requires a minimum ISI that is about 1 ms longer than the latency of the N20 component of the somatosensory evoked potential, and can be obtained over a range of ISIs of N20 + 7–8 ms. Epidural recordings have demonstrated the intracortical origin of SAI (Tokimura et al., 2000). As with other forms of paired-pulse inhibition, the late I-waves are suppressed, whilst the I1 wave is unaffected. According to the canonical microcircuit model, SAI might be produced by excitatory thalamic inputs to GABAAergic cells projecting upon corticospinal cells. Thus, the enhancement of the excitability of these inhibitory interneurons can explain the selective suppression of late I-waves (Figure 2).

## Conclusion

By incorporating the known physiology of the corticospinal multiple discharge evoked by single or paired TMS into the anatomical and computational characteristics of the canonical microcircuit model of neocortex, composed of layer II and III and layer V excitatory pyramidal cells, inhibitory interneurons, and cortico-cortical and thalamo-cortical inputs, the main characteristics of the D- and I-waves, including their regular and rhythmic nature, their stimulus intensity-dependence and their pharmacological modulation can be elegantly and sparsely explained. A TMS-induced strong depolarization of the superficial excitatory cells of the canonical microcircuit may lead to the recruitment of fully synchronized clusters of excitatory neurons, including layer V PTNs, and inhibitory neurons producing a high frequency (~670 Hz) repetitive discharge of the corticospinal axons. The role of the inhibitory circuits is crucial to entrain and control the firing of the excitatory networks to produce a high frequency discharge (Douglas et al., 1989).

It should be considered, however, that the attempt to explain the physiological basis of TMS using the canonical cortical circuit has several major limitations because the model used in this study is extremely simplistic. It should be considered that the canonical circuit we adopted is composed of a minimum of elements and connections and thus it can capture only the essence of the function of cerebral cortex. The interaction between TMS and cerebral cortex is much more complex in that there is a great number of classes of cortical neurons and connections that can be activated by TMS but were not considered in the present paper. Moreover, this simple model cannot easily been used to explain the interaction between TMS and cortical circuits in pathological conditions characterized by structural or functional changes in cerebral cortex.

### Conflict of interest statement

The authors declare that the research was conducted in the absence of any commercial or financial relationships that could be construed as a potential conflict of interest.
